# Correction: The effect of a harmful algal bloom (*Karenia selliformis*) on the benthic invertebrate community and the sea otter (*Enhydra lutris*) diet in eastern Hokkaido

**DOI:** 10.1371/journal.pone.0336854

**Published:** 2025-11-13

**Authors:** Jackson Johnstone, Ippei Suzuki, Randall William Davis, Natsuki Konno, Kyohei Murayama, Satsuki Ochiai, Yoko Mitani

In Figs 2 and 3, the panel labels embedded in the images are missing. Please see the correct Figs 2 and 3 here.

**Fig 2:**
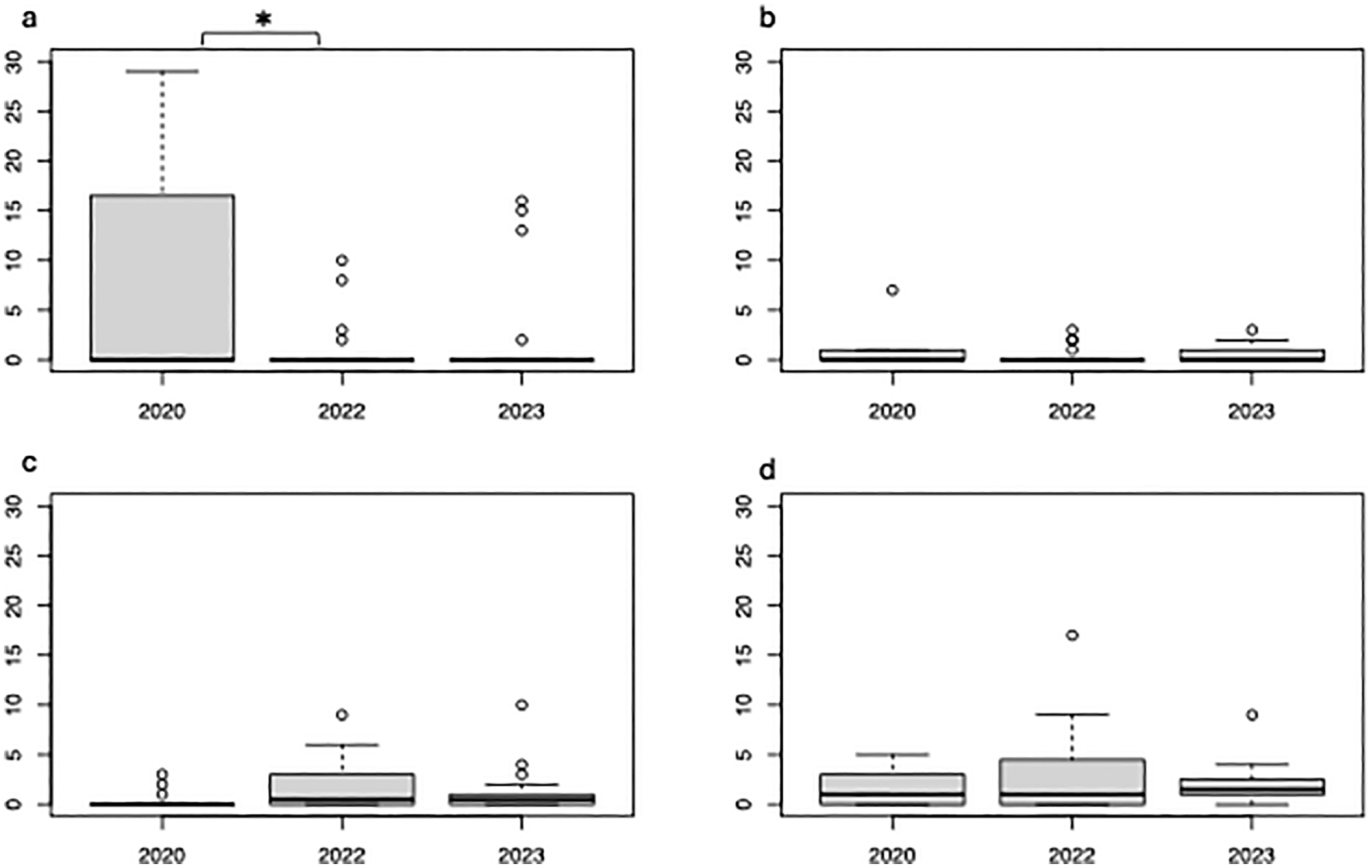
Number (per m²) of major sea otter prey items sampled from SCUBA survey: *a* (Sea Urchins), *b* (Chitons), *c* (Bivalves), *d* (Snails) retrieved during benthic quadrat surveys of 2020, 2022, and 2023. Asterisks (*) above the graph display a significant difference (p < 0.05) between two years.

**Fig 3:**
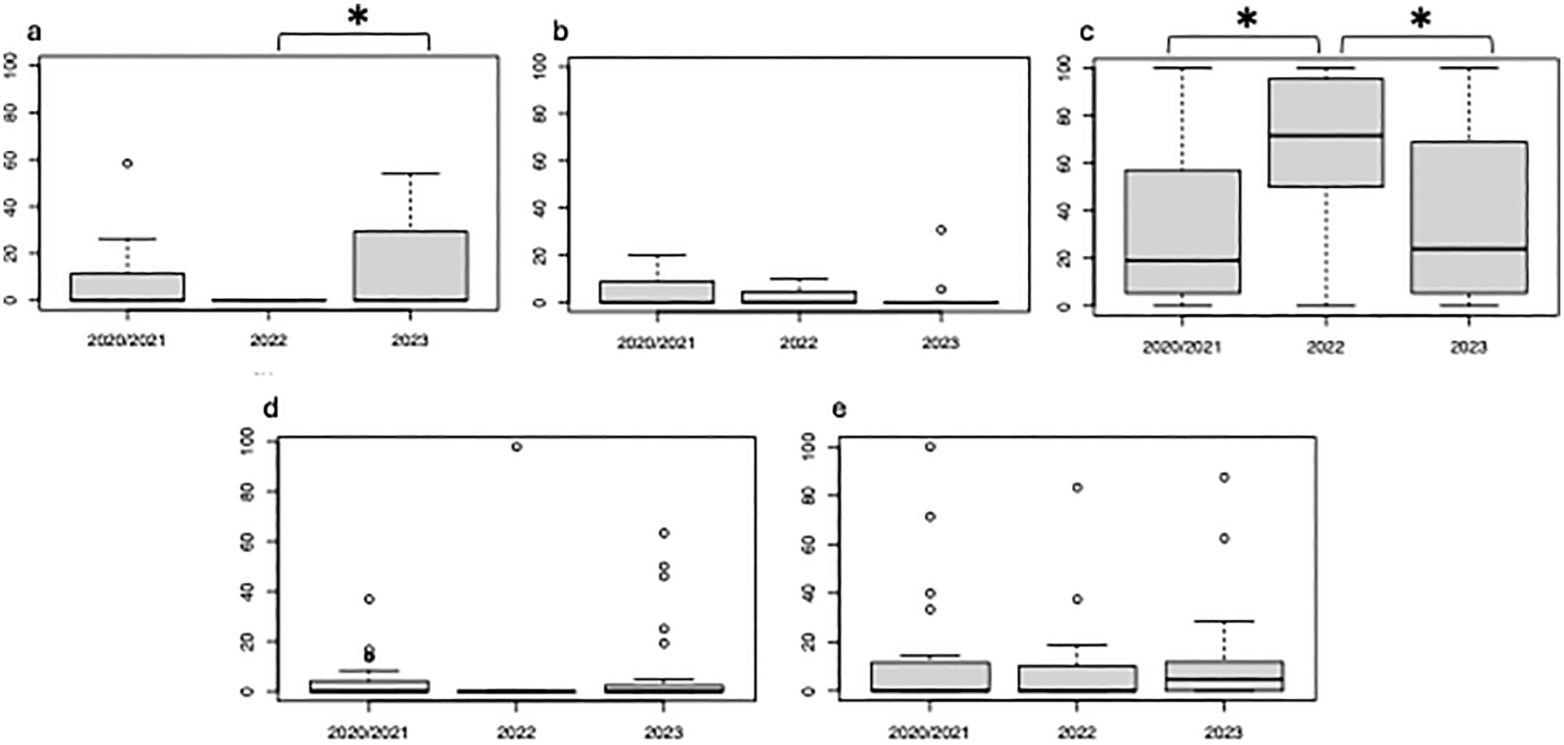
Observed average proportion of major prey items of sea otters: *a* (Sea Urchins), *b* (Chitons), *c* (Bivalves), *d* (Snails), and *e* (Crabs) per bout in the Sea Otter diets at Moyururi and Yururi Islands from 2020/ 2021, 2022, and 2023. *Error bars* represent ±1 SE. Asterisks (*) above the graph display a significant difference (p < 0.05) between two or more years.
