# Supplementary material for: The effect of a harmful algal bloom (Karenia selliformis) on the benthic invertebrate community and the sea otter (Enhydra lutris) diet in eastern Hokkaido
Source: PLoS One. 2024 Nov 21;19(11):e0303126. doi: 10.1371/journal.pone.0303126 (PMC11581392; doi:10.1371/journal.pone.0303126)
Supplement: S1 Appendix — The total number of small, medium, and large bivalves (a) collected during SCUBA dive surveys conducted in September 2020, 2022, and 2023. Number (per m²) of b (small), c (medium), and d (large) bivalves retrieved during benthic quadrat surveys of 2020,2022 and 2023. Asterisks (*) above the graph display a significant difference (p < 0.05) between two years. (DOCX) [file pone.0303126.s001.docx]

*d*

*b*

*c*

*a*

**S1 Appendix** The total number of small, medium, and large bivalves (*a*) collected during SCUBA dive surveys conducted in September 2020, 2022, and 2023. Number (per m²) of *b* (small), *c* (medium), and *d* (large) bivalves retrieved during benthic quadrat surveys of 2020,2022 and 2023. Asterisks (*) above the graph display a significant difference (p < 0.05) between two years.
