# Supplementary material for: The effect of a harmful algal bloom (Karenia selliformis) on the benthic invertebrate community and the sea otter (Enhydra lutris) diet in eastern Hokkaido
Source: PLoS One. 2024 Nov 21;19(11):e0303126. doi: 10.1371/journal.pone.0303126 (PMC11581392; doi:10.1371/journal.pone.0303126)
Supplement: S1 File — (PDF) [file pone.0303126.s002.pdf]

	2020						
Points	Sea Urchin	Sea Cucumbe	Chiton	Crab	Hermit Crab	Bivalve	Snail
16	2	0	0	0	0	3	1
17	0	0	0	1	0	0	0
18	0	0	0	0	0	0	1
19	18	10	1	0	0	0	4
15	0	0	0	0	0	0	1
22	0	0	0	1	0	0	0
21	0	0	0	0	0	0	0
20	0	1	0	0	0	0	0
14	0	0	0	0	1	0	0
13	0	0	0	1	0	2	5
10	16	2	0	0	0	0	0
11	0	1	0	0	1	0	0
12	0	3	1	3	0	0	2
9	5	1	1	0	0	0	0
8	17	0	0	0	0	1	1
7	29	12	1	0	0	0	3
3	0	1	2	4	0	0	0
4	0	0	0	0	0	0	0
6	0	0	0	0	0	1	5
5	19	2	7	0	0	0	3
2	28	3	1	0	2	0	1
1	17	5	0	0	11	0	4
SUM	134	40	12	6	15	6	30
Avg Per m^2	6.7	2	0.6	0.3	0.75	0.3	1.5

Note: Order of points was not relevant for analysis

Points 3 and 8 were not included in analysis due to the research team
not being able to access those sites in 2023

Note: Order of points was not relevant for analysis

Points 3 and 8 were not included in analysis due to the research team not being able to acce

[redacted]

	2022						
Point	Sea Urchin	Sea Cucumbe	Chiton	Crab	Hermit Crab	Bivalve	Snail
1	8	2	1	0	13	0	5
2	10	1	0	0	3	0	4
3	0	0	0	0	1	0	0
4	0	0	0	0	4	0	1
5	3	3	2	4	0	0	17
6	0	0	0	0	0	1	0
7	2	2	2	1	6	2	9
8	2	0	2	1	8	3	0
9	0	0	0	0	0	0	2
10	0	0	3	0	0	3	0
11	0	0	0	0	1	3	1
12	0	0	0	0	1	3	0
13	0	0	0	0	2	4	8
14	0	0	0	0	1	6	1
15	0	0	0	0	0	3	0
16	0	0	0	0	1	9	0
17	0	0	0	0	4	0	7
18	0	0	0	0	0	0	0
19	0	0	0	1	0	1	0
20	0	1	0	0	0	0	1
21	0	0	0	0	1	0	0
22	0	0	0	0	20	0	0
SUM	23	9	8	6	57	35	56
Avg Per m^2	1.15	0.45	0.4	0.3	2.85	1.75	2.8

ss those sites in 2023

[redacted]

	2023								
Points	Station	Sea Urchin	Sea Cucumbe	Chiton	Crab	Sea Star	Hermit Crab	Bivalve	Snail
1	22	13	7	0	0	3	2	0	1
2	21	15	0	2	0	3	14	0	9
3	17	0	0	0	0	0	0	0	0
4	18	0	0	1	2	0	3	0	2
5	20	16	1	3	3	3	1	0	3
6	19	0	0	1	0	0	0	1	1
7	16	0	0	1	1	0	1	3	2
8	15	0	0	0	0	0	0	0	0
9	14	0	0	1	5	0	5	1	1
10	11	0	2	0	0	0	0	2	0
11	12	0	0	0	0	1	3	1	2
12	13	0	0	1	2	2	4	0	1
13	10	0	0	0	1	0	2	1	1
14	9	0	0	0	0	0	2	4	0
15	5	2	0	0	0	0	2	10	2
16	1	0	0	0	0	0	2	1	0
17	2	0	0	0	0	0	6	0	4
18	3	0	0	0	2	0	0	0	3
19	4	0	0	0	0	0	0	0	2
20	8	0	0	0	0	0	2	0	3
21	7	0	0	0	0	0	0	0	1
22	6	0	0	0	0	0	0	1	1
SUM	221	46	10	10	16	12	49		
Avg Per m^2	11.05	2.3	0.5	0.5	0.8	0.6	2.45		

2020

Points	Large Bivalve	Medium Biva	Small Bivalves
16	1	1	1
17	0	0	0
18	0	0	0
19	0	0	0
15	0	0	0
22	0	0	0
21	0	0	0
20	0	0	0
14	0	0	0
13	0	2	0
10	0	0	0
11	0	0	0
12	0	0	0
9	0	0	0
8	0	1	0
7	0	0	0
3	0	0	0
4	0	0	0
6	0	0	1
5	0	0	0
2	0	0	0
1	0	0	0

[redacted]

2022

Point	Large Bivalve	Medium Biva	Small Bivalves
1			
2			
4			
5			
6		1	
7			2
9			
10		1	2
11		3	
12			3
13			4
14		4	2
15	3		
16	2		7
17			
18			
19			1
20			
21			
22			

[redacted]

Blank symbolizes zero in these columns

2023

Points	Station	Large Bivalve	Medium Biva	Small Bivalves
1	22			
2	21			
4	18			
5	20			
6	19			1
7	16		2	1
9	14		1	
10	11	1	1	
11	12			1
12	13			
13	10		1	
14	9		4	
15	5	4	1	2
16	1	1		
17	2			
18	3			
19	4			
20	8			
21	7			
22	6	1		

2020/2021

ing Dive Nu	al Successfu Di	Total# preys	Sea Urchin	Sea Cucumber	Chiton	Crab	Bivalve	Snail	nknown Shellfi	nown Non She	Unknown Tota	% Sea Urchins	Sea Cucumbe	% Chitons	Crab	% Bivalve	% Snail	% Unknown
FDG8_2020_	7	12	0	0	0	0	0	2	0	10	10	0	0	0	0	0	16.6666667	83.3333333
FDG12_2020	7	7	0	0	1	5	0	0	0	1	1	0	0	14.2857143	71.4285714	0	0	14.2857143
FDG14_2020	10	12	1	0	0	0	11	0	0	0	0	8.33333333	0	0	0	91.6666667	0	0
FDG15_2020	9	13	0	0	0	0	4	0	0	9	9	0	0	0	0	30.7692308	0	69.2307692
FDG20_2020	10	11	0	0	0	1	2	0	1	7	8	0	0	0	9.09090909	18.1818182	0	72.7272727
FDG21_2020	8	12	2	0	0	4	2	1	0	3	3	16.6666667	0	0	33.3333333	16.6666667	8.33333333	25
FDG22_2020	5	5	0	0	0	2	0	0	0	3	3	0	0	0	40	0	0	60
FDG23_2020	6	9	2	0	0	0	7	0	0	0	0	22.2222222	0	0	0	77.7777778	0	0
FDG24_2020	6	7	1	0	1	1	3	0	0	1	1	14.2857143	0	14.2857143	14.2857143	42.8571429	0	14.2857143
FDG29_2020	6	8	0	0	0	0	8	0	0	0	0	0	0	0	0	100	0	0
FDG32_2020	8	15	0	0	0	0	7	2	0	6	6	0	0	0	0	46.6666667	13.3333333	40
FDG34_2020	6	8	0	0	0	0	0	0	2	6	8	0	0	0	0	0	0	100
FDG36_2020	8	10	0	0	1	0	5	0	0	4	4	0	0	10	0	50	0	40
FDG37_2021	6	6	0	0	0	6	0	0	0	0	0	0	0	0	100	0	0	0
FDG38_2021	8	14	1	0	0	1	9	2	0	1	1	7.14285714	0	0	7.14285714	64.2857143	14.2857143	7.14285714
FDG43_2021	10	23	1	0	0	0	22	0	0	0	0	4.34782609	0	0	0	95.6521739	0	0
FDG46_2021	5	5	0	0	1	0	1	0	1	2	3	0	0	20	0	20	0	60
FDG47_2021	9	13	0	0	1	0	2	0	10	0	10	0	0	7.69230769	0	15.3846154	0	76.9230769
FDG48_2021	10	19	5	0	2	0	2	7	0	3	3	26.3157895	0	10.5263158	0	10.5263158	36.8421053	15.7894737
FDG50_2021	6	12	7	0	0	0	2	0	3	0	3	58.3333333	0	0	0	16.6666667	0	25
											Unweighted	7.88238713	0	3.8395026	13.7640693	34.8550728	4.47305764	35.1859106
											Unweighted	14.4508478	0	6.4207828	27.4934505	34.4364493	9.42074268	32.8803932

Feeding Dives Were Selected from Dataset according to criteria specified in materials and methods

[redacted]

FDG#	Total Succes	Total# preys	Sea Urchin	Sea Cucumbe	Chiton	Crab	Bivalve	Snail	Unknown Sh	Unknown No	Unknown Tot	% Sea Urchi	% Sea Cucum	% Chitons	%Crab	% Bivalve	% Snail	% Unknown
FDG51_2022	5	10	0	0	0	0	10	0	0	0	0	0	0	0	0	100	0	0
FDG52_2022	7	10	0	0	1	0	9	0	0	0	0	0	0	10	0	90	0	0
FDG53_2022	10	22	0	0	0	0	22	0	0	0	0	0	0	0	0	100	0	0
FDG54_2022	8	22	0	0	1	0	21	0	0	0	0	0	0	4.54545455	0	95.4545455	0	0
FDG55_2022	6	15	0	0	1	0	14	0	0	0	0	0	0	6.66666667	0	93.3333333	0	0
FDG56_2022	6	10	0	0	0	1	7	0	0	2	2	0	0	0	10	70	0	20
FDG58_2022	10	16	0	1	0	3	11	0	0	1	1	0	6.25	0	18.75	68.75	0	6.25
FDG62_2022	8	15	0	0	1	0	11	0	0	3	3	0	0	6.66666667	0	73.3333333	0	20
FDG65_2022	10	12	0	0	0	0	2	0	0	10	10	0	0	0	0	16.6666667	0	83.3333333
FDG66_2022	9	16	0	0	0	6	8	0	0	2	2	0	0	0	37.5	50	0	12.5
FDG67_2022	10	88	0	0	0	0	0	86	0	2	2	0	0	0	0	0	97.7272727	2.27272727
FDG69_2022	10	20	0	0	0	0	20	0	0	0	0	0	0	0	0	100	0	0
FDG71_2022	10	25	0	0	0	0	17	0	0	8	8	0	0	0	0	68	0	32
FDG72_2022	6	6	0	0	0	5	1	0	0	0	0	0	0	0	83.3333333	16.6666667	0	0
											Unweighted	0	0.44642857	1.99134199	10.6845238	67.3003247	6.98051948	12.5968615
											Unweighted	0	1.67038276	3.44220239	23.540522	34.1894106	26.1187123	22.7822289

[redacted]

FDG#	Total Succes	Total# preys	Sea Urchin	Sea Cucumbe	Chiton	Crab	Bivalve	Snail	Hermit Crabs	Unknown Sh	Unknown No	Unknown To	% Sea Urchir	% Sea Cucum	% Chitons	%Crab	% Bivalve	% Snail	% Hermit Cra	% Unknown
FDG80_2023	6	7	0	0	0	2	5	0	0	0	0	0	0	0	0	28.5714286	71.4285714	0	0	0
FDG81_2023	8	13	7	0	4	1	0	0	0	0	1	1	53.8461538	0	30.7692308	7.69230769	0	0	0	7.69230769
FDG82_2023	9	18	9	0	0	1	5	0	0	0	3	3	50	0	0	5.55555556	27.7777778	0	0	16.6666667
FDG83_2023	10	21	2	0	0	0	12	1	0	0	6	6	9.52380952	0	0	0	57.1428571	4.76190476	0	28.5714286
FDG84_2023	7	8	0	0	0	7	0	0	0	0	1	1	0	0	0	87.5	0	0	0	12.5
FDG85_2023	8	23	12	0	0	0	6	0	0	0	5	5	52.173913	0	0	0	26.0869565	0	0	21.7391304
FDG87_2023	10	46	18	0	0	0	11	0	0	0	17	17	39.1304348	0	0	0	23.9130435	0	0	36.9565217
FDG89_2023	9	19	0	0	0	4	2	0	0	0	13	13	0	0	0	21.0526316	10.5263158	0	0	68.4210526
FDG90_2023	8	16	0	0	0	0	16	0	0	0	0	0	0	0	0	0	100	0	0	0
FDG91_2023	9	24	0	0	0	0	0	6	0	0	18	18	0	0	0	0	0	25	0	75
FDG93_2023	8	21	9	0	0	2	3	0	0	0	7	7	42.8571429	0	0	9.52380952	14.2857143	0	0	33.3333333
FDG96_2023	6	26	8	0	0	1	0	5	0	0	12	12	30.7692308	0	0	3.84615385	0	19.2307692	0	46.1538462
FDG99_2023	8	8	1	0	0	5	1	0	0	0	1	1	12.5	0	0	62.5	12.5	0	0	12.5
FDG104_202	10	18	1	0	1	2	3	0	0	0	11	11	5.55555556	0	5.55555556	11.1111111	16.6666667	0	0	61.1111111
FDG105_202	9	16	0	0	0	1	15	0	0	0	0	0	0	0	0	6.25	93.75	0	0	0
FDG106_202	8	13	0	0	0	2	11	0	0	0	0	0	0	0	0	15.3846154	84.6153846	0	0	0
FDG107_202	10	24	0	0	0	0	24	0	0	0	0	0	0	0	0	0	100	0	0	0
FDG111_202	7	15	0	0	0	0	14	0	0	0	1	1	0	0	0	0	93.3333333	0	0	6.66666667
FDG116_202	10	37	0	0	0	1	4	17	4	0	11	11	0	0	0	2.7027027	10.8108108	45.9459459	10.8108108	29.7297297
FDG117_202	10	49	0	0	0	1	0	31	3	0	14	14	0	0	0	2.04081633	0	63.2653061	6.12244898	28.5714286
FDG119_202	5	18	5	0	0	0	12	0	0	0	1	1	27.7777778	0	0	0	66.6666667	0	0	5.55555556
FDG121_202	10	29	3	0	0	0	19	0	0	0	7	7	10.3448276	0	0	0	65.5172414	0	0	24.137931
FDG122_202	10	25	0	0	0	2	6	0	2	0	15	15	0	0	0	8	24	0	8	60
FDG_123_20	6	24	0	0	0	3	0	12	1	0	8	8	0	0	0	12.5	0	50	4.16666667	33.3333333
												Unweighted	13.9366186	0	1.51353276	11.8429638	37.4592225	8.67516359	1.21249694	25.3600018
												Unweighted	19.7055831	0	6.33359231	21.1437704	37.0389637	18.4447754	2.9520483	23.0505846

Benthic Sea Urchin Numbers

Year	Number Per M^2
2020	2
2020	0
2020	0
2020	18
2020	0
2020	0
2020	0
2020	0
2020	0
2020	0
2020	16
2020	0
2020	0
2020	5
2020	29
2020	0
2020	0
2020	19
2020	28
2020	17
2022	8
2022	10
2022	0
2022	3
2022	0
2022	2
2022	0
2022	0
2022	0
2022	0
2022	0
2022	0
2022	0
2022	0
2022	0
2022	0
2022	0
2022	0
2022	0
2022	0
2023	13
2023	15
2023	0
2023	16
2023	0
2023	0
2023	0
2023	0
2023	0
2023	0
2023	0
2023	0
2023	2
2023	0
2023	0
2023	0
2023	0
2023	0
2023	0
2023	0

Percent of sea urchins within sea otter diet

Year	Percent
2020/2021	0
2020/2021	0
2020/2021	8.33333333
2020/2021	0
2020/2021	0
2020/2021	16.6666667
2020/2021	0
2020/2021	22.2222222
2020/2021	14.2857143
2020/2021	0
2020/2021	0
2020/2021	0
2020/2021	0
2020/2021	0
2020/2021	7.14285714
2020/2021	4.34782609
2020/2021	0
2020/2021	0
2020/2021	26.3157895
2020/2021	58.3333333
2022	0
2022	0
2022	0
2022	0
2022	0
2022	0
2022	0
2022	0
2022	0
2022	0
2022	0
2022	0
2022	0
2022	0
2023	0
2023	53.8461538
2023	50
2023	9.52380952
2023	0
2023	52.173913
2023	39.1304348
2023	0
2023	0
2023	0
2023	42.8571429
2023	30.7692308
2023	12.5
2023	5.55555556
2023	0
2023	0
2023	0
2023	0
2023	0
2023	0
2023	27.7777778
2023	10.3448276
2023	0
2023	0
