# Supplementary material for: The effect of a harmful algal bloom (Karenia selliformis) on the benthic invertebrate community and the sea otter (Enhydra lutris) diet in eastern Hokkaido
Source: PLoS One. 2024 Nov 21;19(11):e0303126. doi: 10.1371/journal.pone.0303126 (PMC11581392; doi:10.1371/journal.pone.0303126)
Supplement: S2 File — (DOCX) [file pone.0303126.s003.docx]

R code sample

**# For Benthic Organism Survey**

**# Example: Sea Urchins**

Setwd(----)

a <- read.table("----", h=T, sep="\t")

a$Year <- as.factor(a$Year)

a$Number_PerMSq <- as.numeric(a$Number_PerMSq)

str(a)

shapiro.test(a$Number_PerMSq)

boxplot(a$Number_PerMSq~a$Year,ylim=c(0,30), notch=F)

summary(fm1 <- aov(a$Number_PerMSq~a$Year, a = a))

TukeyHSD(fm1, ordered = TRUE)

**# For Feeding Dive Analysis**

**# Example: Sea Urchins/ SU**

Setwd(----)

a <- read.table(----, h=T, sep="\t")

a$Year <- as.factor(a$Year)

a$SUpct <- as.numeric(a$SUpct)

str(a)

shapiro.test(a$SUpct)

boxplot(a$SUpct~a$Year,ylim=c(0,100), notch=F)

summary(fm1 <- aov(a$SUpct~a$Year, a = a))

TukeyHSD(fm1, ordered = TRUE)

For Bivalve Size Classes

Ex: Small Bivalves

Setwd(----)

a <- read.table("------", h=T, sep="\t")

a$Year <- as.factor(a$Year)

a$Number_PerMSq <- as.numeric(a$Number_PerMSq)

str(a)

shapiro.test(a$Number_PerMSq)

boxplot(a$Number_PerMSq~a$Year,ylim=c(0,25), notch=F)

summary(fm1 <- aov(a$Number_PerMSq~a$Year, a = a))

TukeyHSD(fm1, ordered = TRUE)
