# Supplementary material for: The effect of a harmful algal bloom (Karenia selliformis) on the benthic invertebrate community and the sea otter (Enhydra lutris) diet in eastern Hokkaido
Source: PLoS One. 2024 Nov 21;19(11):e0303126. doi: 10.1371/journal.pone.0303126 (PMC11581392; doi:10.1371/journal.pone.0303126)
Supplement: Attachment — Submitted filename: Response to Reviewers.docx [file pone.0303126.s004.docx]

Response to editor revisions:

1. Please ensure that your manuscript meets PLOS ONE's style requirements, including those for file naming.

-Thank you very much for your comments. I have reformatted the titles for the documents in my submission.

2. Thank you for stating the following financial disclosure: 
"This study was funded by the Asahi Glass Foundation ([https://www.af-info.or.jp/en/research/](https://www.af-info.or.jp/en/research/)) in 2020-2021 (to I.S. and Y.M.), the Sasakawa Peace Foundation ([https://www.spf.org/en/](https://www.spf.org/en/)) in 2021 (to S.O. and Y.M.), the Hokkaido University DX Doctoral Fellowship ([https://sites.google.com/eis.hokudai.ac.jp/dxphd-fellow/home](https://sites.google.com/eis.hokudai.ac.jp/dxphd-fellow/home)) for 2022-2023 (to J.J.), the Pro Natura Fund ([https://www.pronaturajapan.com/en/index.html](https://www.pronaturajapan.com/en/index.html)) (to I.S., R.D., and Y.M.), and the JSPS (Japanese Society for the Promotion of Science) Invitational Fellowships for Research in Japan ([https://www.jsps.go.jp/english/e-inv/](https://www.jsps.go.jp/english/e-inv/)) (to R.D. and Y.M.)."

Please state what role the funders took in the study.  If the funders had no role, please state: ""The funders had no role in study design, data collection and analysis, decision to publish, or preparation of the manuscript."" 
If this statement is not correct you must amend it as needed. 
Please include this amended Role of Funder statement in your cover letter; we will change the online submission form on your behalf.

-I have added a section that includes the lack of a role for the funders from the study construction in the Cover letter.

3. Thank you for stating the following in the Acknowledgments Section of your manuscript: 
"This study was funded by the Asahi Glass Foundation in 2020-2021 (to I.S. and Y.M.), the Sasakawa Peace Foundation in 2021 (to S.O. and Y.M.), the Hokkaido University DX Doctoral Fellowship for 2022-2023 (to J.J.), the Pro Natura Fund (to I.S., R.D., and Y.M.), and the JSPS (Japanese Society for the Promotion of Science) Invitational Fellowships for Research in Japan (to R.D. and Y.M.)."

We note that you have provided funding information that is not currently declared in your Funding Statement. However, funding information should not appear in the Acknowledgments section or other areas of your manuscript. We will only publish funding information present in the Funding Statement section of the online submission form. 
Please remove any funding-related text from the manuscript and let us know how you would like to update your Funding Statement. Currently, your Funding Statement reads as follows: 
"This study was funded by the Asahi Glass Foundation ([https://www.af-info.or.jp/en/research/](https://www.af-info.or.jp/en/research/)) in 2020-2021 (to I.S. and Y.M.), the Sasakawa Peace Foundation ([https://www.spf.org/en/](https://www.spf.org/en/)) in 2021 (to S.O. and Y.M.), the Hokkaido University DX Doctoral Fellowship ([https://sites.google.com/eis.hokudai.ac.jp/dxphd-fellow/home](https://sites.google.com/eis.hokudai.ac.jp/dxphd-fellow/home)) for 2022-2023 (to J.J.), the Pro Natura Fund ([https://www.pronaturajapan.com/en/index.html](https://www.pronaturajapan.com/en/index.html)) (to I.S., R.D., and Y.M.), and the JSPS (Japanese Society for the Promotion of Science) Invitational Fellowships for Research in Japan ([https://www.jsps.go.jp/english/e-inv/](https://www.jsps.go.jp/english/e-inv/)) (to R.D. and Y.M.)."

-I have removed this statement from the acknowledgments section of my manuscript and would like to include the following at the bottom of a revised cover letter file:

**Funding: This study was funded by the Asahi Glass Foundation ([https://www.af-info.or.jp/en/research/](https://www.af-info.or.jp/en/research/)) in 2020-2021 (to I.S. and Y.M.), the Sasakawa Peace Foundation ([https://www.spf.org/en/](https://www.spf.org/en/)) in 2021 (to S.O. and Y.M.), the Hokkaido University DX Doctoral Fellowship ([https://sites.google.com/eis.hokudai.ac.jp/dxphd-fellow/home](https://sites.google.com/eis.hokudai.ac.jp/dxphd-fellow/home)) for 2022-2023 (to J.J.), the Pro Natura Fund ([https://www.pronaturajapan.com/en/index.html](https://www.pronaturajapan.com/en/index.html)) (to I.S., R.D., and Y.M.), and the JSPS (Japanese Society for the Promotion of Science) Invitational Fellowships for Research in Japan ([https://www.jsps.go.jp/english/e-inv/](https://www.jsps.go.jp/english/e-inv/)) (to R.D. and Y.M.).**

**Role of funder: The funders had no role in study design, data collection, and analysis, the decision to publish, or the preparation of the manuscript.**

4. Please include your full ethics statement in the ‘Methods’ section of your manuscript file. In your statement, please include the full name of the IRB or ethics committee who approved or waived your study, as well as whether or not you obtained informed written or verbal consent. If consent was waived for your study, please include this information in your statement as well.

-I have included the ethics statement as point 5 of my materials and methods section of the manuscript. I have also included the code of conduct established by the Kyoto University Wildlife Research Center that we followed during the course of our research (168-176).

5. Please ensure that you refer to Figure 1 in your text as, if accepted, production will need this reference to link the reader to the figure.

-I have added a reference within text to figure 1 in the study area point of the materials and methods section of the revised manuscript (Line 109).

6. Please include a separate caption for each figure in your manuscript.

-Each figure has a title/ caption

7. Please include your tables as part of your main manuscript and remove the individual files. Please note that supplementary tables (should remain/ be uploaded) as separate ""supporting information"" files

-I have included the tables within the revised manuscript, in lines 191-194 and 208-211.

8. Please include captions for your Supporting Information files at the end of your manuscript, and update any in-text citations to match accordingly. Please see our Supporting Information guidelines for more information: [http://journals.plos.org/plosone/s/supporting-information](http://journals.plos.org/plosone/s/supporting-information).

-Supporting information caption is now included at the end of the manuscript.

Reviewer’s comments section:

Reviewer #1: The authors are to be commended for the work presented in this manuscript. Information about the biology and natural history of Hokkaido's sea otter population is scarce, so any additions are important assets to sea otter conservation. Overall, I feel that this paper is well written, contains important information, and I only have a few suggestion for minor edits.

-Thank you very much for your kind words and comments and suggestions.

The overall purpose of this study and subsequent manuscript is not clearly stated. I recommend that the authors revisit how the purpose is described to ensure that it is clear to the reader.

-I have tried to add more clarity to the purpose of the experiment towards the end of my introduction section, lines 97-105. I reemphasized the novelty of this research, in how it offers one of the first opportunities to observe this population and a unique opportunity to observe the effects of a red tide upon it. I also directly stated what our research seeks to do (our purpose) to go with what we already stated were the goals of the research.

Line 100-111: I believe that the benthic survey methodology is valid, but the methods section would be strengthened by including reference to similar efforts that have already been through review and published.

-The survey methodology is taken from the same paper (Kvitek et al., 1993) that we followed for designing the protocol for deciding benthic organism side classes. I have included another reference to the paper on line 116. The Kvitek paper is foundational to the field of sea otter dietary preference in regards to the availability of prey items.

Lines 41-42 & 208-209: The authors should expand their discussion on how they came to the conclusion that there were no health effects on the otters.

-The existing line about the population remaining unchanged provides the evidence we have that there were no observed negative health effects on the population. I am using the yet-unpublished research data (Suzuki et al., in prep) as evidence for this lack of change.
